# 

**Published:** 1892-08-13

**Authors:** 


**Notices of Births, Deaths, and Marriages,** are inserted in this column at a charge of 2s. 6d. for an announcement not exceeding four lines.

### NOTICE TO CORRESPONDENTS.

All MS., Letters, Books for Review, and other matters intended for the Editor should be addressed The Editor, The Lodge, Porchester Square, London, W.

The Editor cannot undertake to return rejected M.S., even when accompanied by stamped directed envelope.

**Notice to Advertisers and Subscribers.**

All Advertisements, Orders for Copies of The Hospital, and Business Communications should be addressed to The Publisher, *not to the Editor*, The Hospital, 140, Strand, W.C.

The Cost of Subscription, post free, is as follows:—Per week, 2½d.; per quarter, 2s. 6d.; per half-year, 5s.; per annum, 8s. 6d. Foreign:—Per annum, 10s. 8d.; payable, in all cases, in advance.

**"Burdett's Hospital Annual," 1891—1892.**

Third year of publication. 600 pp. Boards, gilt lettering. A most complete guide to British and Colonial Hospitals, Dispensaries, Nursing and Convalescent Institutions, and Asylums. Price 3s. 6d. Published by The Scientific Press (Limited), 140, Strand, W.C.

**NOTICE.—The Index for Vol. XI. (ending March, 1892) is on sale at the Office of this Journal, price 2½d., post free.**

### SCRAPS AND GLEANINGS.

The total sum collected on Hospital Saturday at Brighton amounted to £1,075 18s. 0½d.

The Hospital Saturday collection in the Woolwich district resulted in the receipt of £283 15s. 1d.

Lord Tennyson, who completed his eighty-third year on Saturday last, spent his birthday at his summer residence, Aldworth, on the Blackdown Hills, near Haslemere.

The total subscriptions towards the building fund of the new St. Leonard's Hospital, Bedford, now amount to £602 9s. 8d. It is hoped the new building will be opened in September.

The Robin Society arranged breakfasts last Christmas Day for 10,500 poor London children, it is now asking for funds to enable sickly little ones to be sent into the country for a week's holiday.

Her Majesty has graciously consented to accept the right of placing a child in the Merchant Seamen's Orphan Asylum, Snaresbrook, and has intimated that it may be known as "The Queen's Presentation."

The death is announced of Mr. Samuel Armstrong Lane, the founder and for many years the principal of the School of Medicine adjoining St. George's Hospital, and for many years surgeon of St. Mary's Hospital.

The number of entries for the long-distance ride between Berlin and Vienna, which is to take place on October 1st, is as follows: 333 Austrian officers and 343 German officers. So large an entry was scarcely expected by the promoters of the race.

Earl Spencer has privately sold the Althorp Library for, it is stated, £2 5,000. Mrs. Rylands, of Longford Hall, Manchester, the widow of the millionaire cotton manufacturer, was the purchaser, and she intends to present the library to Manchester.

A band and chorus, 200 strong, under the direction of Mr. Thomas Goulding, performed several hymns and choruses from the "Messiah" on Hospital Sunday at Hoyland. The proceeds amounted to £70, which will be devoted to the hospitals at Barnsley, Sheffield, &c.

The Duke of Westminster has kindly consented to open Grosvenor House on August 14th and 21st from two to six p.m. Tickets may be had by forwarding stamped addressed envelope, stating date and hour for which they are required, to Mr. Henry Mills, Secretary, 221, High Holborn.

A large bazaar and fancy fair in aid of the Training Home for Friendless Girls at Marlborough was held in the beautiful orangery at Savernake House, under the auspices of Lord and Lady Frederick Bruce, the Marchioness of Bath, Lady Doreen Long, the Countess of Craven, and others.

The Oxford Benefit and Trade Societies' Hospital Sunday Fund has been divided thus: £60 to the Radcliffe Infirmary, £15 each to the Sarah Ac and Home and the Eye Hospital, and £5 to the Homœopathic Institution—£95 in all; leaving a balance in hand of £5 odd to meet any unforeseen expenses.

Shelley's centenary was duly celebrated at Horsham, where the poet was born on August 4th, 1792. A memorial tablet to his honour has been placed in the Parish Church. It is also intended to erect in the town a museum and a library, to be called after the poet, for which a national subscription is asked.

The annual report of the Sheffield Public Hospital and Dispensary shows that during the past year 904 in-patients have been treated—an increase of 51 on the previous year. The total expenditure amounted to £5,444 19s. 3d.; the total income was only £4,386 18s. 5d., being a deficiency of about £1,058 0s. 10d.

The returns presented to the officials of the Metropolitan Asylums Board at the end of last week showed that there were 3,244 patients under treatment in the seven hospitals, and of these 2,868 are suffering from scarlet fever, 290 from diphtheria, three from typhus, 78 from enteric fever, and five from other diseases.

The Board of Management of the Derby and Derbyshire Convalescent Home have decided to accept a house at Darley Dale, which has been offered to them by Mr. John Taylor, of Ilkley. Their object is to give a fortnight's holiday to a number of deserving boys and girls. The house will hold sixteen. Subscriptions have already been promised for the furnishing and starting the Home.

The Duchess of Albany met with a most cordial reception on her visit to Godalming, to open the Meath Home of Comfort for Epileptics. Mr. J. P. Whately presented Her Royal Highness with an address on behalf of the Mayor and Corporation of the Borough. The proceedings opened with a Service of Consecration, conducted by the Bishop of Guildford. The Countess of Meath explained the origin and object of the institution, which was enforced by Dr. Russell Reynolds.

At a meeting of the Brompton Hospital for Consumption the following legacies were announced: Miss Cohen, £100; Miss A. Hale, £50; Mr. S. J. Martin, £200; Mr. R. Baxter, £20; Mrs. S. A. Lee, £[illegible]00; Mrs. Rasch, £1,000; and Miss H. Read, £50. The Committee acknowledged, with thanks, an acceptable donation of 500 guineas from Baron Hirsch, to name a ward. The number of in-patients since May 20th was 264, and out-patient cases 2,381.

The new infectious diseases hospital at Wallsend is to comprise four distinct blocks of buildings, the front rising to the height of three stories. The south block is built in pavilion style, and divided into two compartments. The east block consists of spacious laundries, &c., for the disinfection of linen. The north block is also erected in the pavilion style, with six beds for males, and six for females, in addition to rooms set apart for the use of the nurses. A mortuary and ambulance are also provided within the enclosure.

### THE STUDENT OF MEDICINE.

[All communications for this Supplement should be addressed to The Editor of The Hospital, at 140, Strand, with the words, "Students' Column," written in the left hand top corner of the envelope.]

### APPOINTMENTS.

Atkinson, Walter Alexander, M.B., B.S.Durh., appointed Surgeon-Lieutenant to the 1st Surrey (South London) Rifles. Bishop, Henry Draper, L.R.C.P.Lond., M.R.C.S.Eng., appointed House Surgeon to the London Hospital. Dolamere, W. H., M.R.C.P., L.R.C.P., L.D.S., appointed Assistant Dental Surgeon to the Dental Hospital of London. Donagan, A. E., L.D.S., R.C.S.Edin., appointed Dental Surgeon to the Royal Institution for Deaf and Dumb Children, Birmingham. Hill, A. B., M.D.Giessen, L.R.C.P., L.R.C.S, D.P.H.Camb., appointed Public Analyst for the County of Warwickshire. Kauffmann, O. J., M.D.Lond., M.R.C.P., M.R.C.S.. appointed Physician for Out-Patients and Pathologist to the Queen's Hospital, Birmingham. McKinnon, A. A., M.R.C.S.Eng., L.R.C.P.Lond., appointed Assistant House Surgeon to the Royal Albert Hospital, Devonport. Mark, C. F., M.D.Irel., M.R.C.S., appointed Honorary Visiting Surgeon to the Brisbane Hospital, Queensland. Morshead, E. G. A., M.D. Brussels, L.R.C.P.Lond., M.R.C.S., appointed Surgeon to the Loyal St. Mary's Lodge of Oddfellows. Sturge, W. Howard, M.B.Lond., appointed House Physician to the London Hospital. Turner, N. H.. L.R.C.P.Lond., M.R.C.S., appointed Surgeon to the Royal Institution for Deaf and Dumb Children, Birmingham. Warden, Chas., M.D.Aberd., F.R.C.S.Edin., M.R.C.S., appointed Honorary Consulting Surgeon to the Royal Institution for Deaf and Dumb Children, Birmingham. Wilson, T. S., M.B.Edin., M.R.C.P.Lond., M.R.C.S., appointed Honorary Consulting Physician to the Royal Institution for Deaf and Dumb Children, Birmingham.

### VACANCIES.

The following vacancies are announced this week, the amount of salary in each case being given after the name of the institution:

### Resident Medical Officers.

Bury Dispensary Hospital, Bury, Lancashire, House Surgeon, must hold medical and surgical diploma—Salary, £60 per annum.

Eday, Orkney, Resident Medical Officer—Salary, £70 per year with practice.

Kent and Canterbury Hospital, House Surgeon; must be registered medical practitioner and unmarried—Salary £90 first year, with board, rising to £100 second year.

Liverpool Infirmary for Children, Assistant House Surgeon, at the end of this month, for six months—Board and lodging provided in lieu of salary.

Suffolk General Hospital, Bury St. Edmunds, House Surgeon—Salary, £100 per annum.

Wrexham Infirmary and Dispensary, House Surgeon—Salary, £80 per annum, with board, &c.

### Non-Resident Medical Officers.

Hinton District, Sturminster Union, Medical Officer and Public Vaccinator—Salary, £40 per annum, &c.

Manchester Hospital for Consumption and Diseases of the Chest and Throat, Honorary Assistant Medical Officer; doubly qualified.

Thornbury Union, Medical Officer for the Almondsbury District—Salary, £70 per annum, &c.

### PASS LISTS.

### University of Glasgow.

The following candidates have passed the Fourth (Final) Professional Examination for the degrees of M.B. and C.M.:—

(A) Candidates who took Pathology in Fourth Professional Examination: H. C. Anderson, C. J. Babes. T. Barrowman, E. Beck, R. Bishop, J. J. Boyd, W. L. Brown, A. Chalmers, M.A., J. E. Chalmers, D. Charters, J. Cochrane, W. Craik, J. D. Davies, R. G. Dick, T. Divine, J. Fisher, W. Fulton, H. N. Gardiner, J. Gillespie, P. N. Grant, T. Hamilton. D. Harris, W. S. Harrison, W. M. Holmes, R. K. Howat, J. Johnson, H. Kerr, M.A., D. C. Laird, R. Langmuir, M.A., J. Lindsay, M.A., J. B. Littlejohn, E. J. Morris, W. M'N. Muat, P. H. Murray, A. C. M'Arthur, J. M'Glashan, J. A. Macintosh, J. M'Kay, A. J. M'Kechnie, G. M'Lauchlan, P. C. MacRobert, C. O'Neill, W. A. Paterson, R. Ramsey, W. Robertson, L. A. Rowden, T. L. Shields, W. N. Sime, A. R. Smith, C. Stewart. D. R. T. Strong, D. Wallace, A. Watson, J. Watt, J. Whitehouse, C. F. Wylie, A. A. Young, M.A., J. C. Young.

(B) Candidates who passed Pathology in Third Professional Examination: W. H. M. Alexander, J. E. Bow, W. B. Brodie, W. M. Brown, A. P. Campbell, D. B. Campbell, J. Carslaw, M.A., A. Cluckie, J. Cochrane, R. Cook, A. B. Craig, J. R. Dalrymple, J. S. Davies, M.A., J. Don, M.A., W. W. Don, McN. Downie, A. R. Ferguson, A. Finlay, A. Forrest,

### THE STUDENT OF MEDICINE—(*continued*).

M.A., F. F. Frew, A. Graham, R. Guy, J. B. Hartley, Æ. Henderson, E. D. S. Heyliger, H. E. Jones, N. Keith, T. Kirkwood, M.A., T. D. Laird, H. Lawrie, H. C. Marr, R. M'F. Marshall, J. D. R. Monro, M.A., J. Morton, W. C. Murray, J. H. MacArthur, W. M'Call, A. H. M'Cracken, J. M'Donald, M.A., A. S. M'Pherson, W. M'Walter, G. Nicoll, J. Paterson, M.A., W. F. Paton, M.A., W. J. Richard, M.A., J. J. Robb, J. A. Robertson, M. T. Stack, W. Steel, W. C. Steele, J. B. Stevens, R. Taylor, J. Thomson, J. M'D. Vallance, A. Wauchope, C. Wilson, W. S. Young.

Doctors of Medicine.—I. With Commendation: H. Highet, M.B., C.M. (Thesis: The Broncho-pneumonia of Influenza as it Occurs in the Adult Subject); A. G. Park, M.B., C.M. (Thesis: Defects of Speech). II. Ordinary Degree.—J. Duncan, M.B., C.M. (Thesis: An Outbreak of Typhoid Fever; its Clinical Features and Treatment); P. Fraser, M.B. (Thesis: An Analysis of Ten Years' Medical Certificates of the Cause of Death); R. A. Morton, B.Sc., M.B., C.M. (Thesis: Hypnotism, with Special Reference to its Phenomena as manifested in Hysterical Subjects, and its Application as a Therapeutic Agent); J. Muir, M.B., C.M. (Thesis: Diphtheria; its Pathology and Therapeutics as primarily a Local Disease); J. F. Muir, M.B., C.M. (Thesis: Typhus Fever); J. S. M'Conville, M.A., M.B., C.M. (Thesis: Chloroform Anæsthesia); A. Shanks, M.B., C.M. (Thesis: Epilepsy; its Symptoms, Causes, and Modern Treatment); J. P. Simpson, M.B., C.M. (Thesis: Puerperal Eclampsia, with Illustrative Cases); J. Somerville, M.B., C.M. (Thesis: Sheffield Grinders' Disease); W. C. Taylor, M.B., C.M. (Thesis: Observations on Small-pox and Vaccination, with Cases); A. S. Tindal, M.B., C.M. (Thesis: The Treatment of Insomnia in Fevers).

Doctor of Medicine (Old Regulations).—R. Cook, L.R.C.S.Ed. (Thesis: The Abortive Treatment of Gonorrhœa).

Bachelors of Medicine and Masters of Surgery.—I. Honours: *J. Morton, J. Carslaw, M.A. II. High Commendation.—J. M'Donald, M.A., J. Paterson, M.A., C. Wilson, R. Bishop, A. R. Ferguson, J. D. R. Monro, M.A., W. B. Brodie. III. Commendation: W. M. Holmes, R. Taylor, J. Don, M.A., H. C. Marr, W. M'Walter, A. A. Young, M.A., Hugh Kerr, M.A., W. L. Brown, W. J. Richard, M.A., W. Roberton, L. A. Rowden. IV. Ordinary Degrees: W. H. M. Alexander, H. C. Anderson, C. J. Babes, T. Barrowman, E. Beck, J. E. Bow, J. J. Boyd, W. M. Brown, A. P. Campbell, D. B. Campbell, A. Chalmers, M.A., J. E. Chalmers, D. Charters, A. Cluckie, James Cochrane, John Cochrane, R. Cook, A. B. Craig, W. Craik, J. R. Dalrymple, J. D. Davies, J. S. Davies, M.A., R. G. Dick, T. Divine, N. Downie, A. Findlay, J. Fisher, A. Forrest, M.A., J. F. Frew, W. Fulton, H. N. Gardiner, J. Gillespie, A. Graham, P. N. Grant, R. Guy, T. Hamilton, D. Harris, J. B. Hartley, Æ. Henderson, E. D. S. Heyliger, R. K. Howat, J. Johnson, H. E. Jones, N. Keith, T. Kirkwood, M.A., D. C. Laird, R. Langmuir, M.A., H. Lawrie, J. Lindsay, M.A., J. B. Littlejohn, R. M'F. Marshall, E. J. Morris, P. H. Murray, W. C. Murray, A. C. M'Arthur, A. H. M'Cracken, J. M'Clashan, J. A. Macintosh, J. M'Kay, A. J. M'Kechnie, G. M'Lauchlan, P. C. MacRobert, C. O'Niell, W. A. Paterson, W. F. Paton, M.A., R. Ramsey, J. J. Robb, W. N. Sime, A. R. Smith, M. T. Stack, W. Steel, W. C. Steele, D. R. T. Strong, J. Thomson, J. M'D. Vallance, D. Wallace, A. Watson, J. Watt, A. Wauchope, J. Whitehouse, C. F. Wylie, J. C. Young.

* Mr. Morton gains the Brunton Memorial Prize of £10, awarded to the most distinguished Graduate in Medicine of the year.

### Examining Board in England by the Royal Colleges of Physicians and Surgeons.

The following gentlemen having passed the necessary examinations have been admitted by the two Royal Colleges Diplomates in Public Health: A. R. Aldridge, M.B.Edin., Edinburgh; H. C. L. Armin (Surgeon-Captain Indian Medical Staff), L.R.C.P., M.R.C.S., Charing Cross; W. H. Bailey, M.B.Lond., M.R.C.S., St. Bartholomew's; W. J. Best, M.R.C.S., London; H. H. Coates, L.R.C.P., M.R.C.S., St. Bartholomew's; J. Gay, L.R.C.P., M.R.C.S., St. Bartholomew's and King's College; J. H. Gibson, M.D.Q.U.I., Dublin and Belfast; J. M. Hill, M.B.R.U.I., Belfast and King's; J. C. Hoyle, L.R.C.P., M.R.C.S., St. Bartholomew's; H. E. Mann, L.R.C.P., M.R.C.S., St. George's; W. Y. Orr, M.B.Edin., Edinburgh and King's; G. Preston, L.R.C.P., M.R.C.S., Manchester; J. D. Priest, M.R.C.S., St. Bartholomew's; T. F. Ricketts, L.R.C.P., M.R.C.S., Guy's; G. I. Schorstein, M.B.Oxon., Oxford and London; S. R. Scott, L.R.C.P., M.R.C.S., Charing Cross; F. Tratman, M.B.Lond., L.R.C.P., M.R.C.S., London and Bristol; J. Wilkie, M.B.Lond., University College and St. Bartholomew's. Eight were referred.

### Royal College of Surgeons, Edinburgh.

The following gentlemen, having passed the requisite examinations, have been admitted Fellows of the College since last publication: Percy John Wilkinson, L.R.C.S.E., 15, Banks Road, West Kirby, Cheshire; William Smyth Crawford, L.R.C.S.E., 40, Rodney Street, Liverpool; James Thomas Moore Giffen, L.R.C.S.E., Thornville, Jordanstown, Belfast; Herbert Andrews Powell, M.R.C.S.Eng., Piccard's Rough, Guildford, and Richard Philip Brooks, M.R.C.S.Eng., 82, Tollington Park, London, N.

The following gentlemen, having passed the requisite examinations, were admitted Licentiates of the College. Andrew Watson Munro, Tain; James Cecil Palmer, New Zealand; Alexander Stuart, Kelso; Lloyd Grant Smith, Cheshire; Pochkhánáválá Dorabjï Hormasjï, Bombay; Charles Scatchard, Yorkshire; and John Edwin Stott, Liverpool.

The following gentlemen, having passed the requisite examinations, were admitted Licentiates in Dental Surgery: Thomas Nash, Edinburgh; Henry Alexander Matheson, Edinburgh; John Malcolm, Edinburgh; and James Joshua Martin, Bradford.

